# Nutritional Ultrasound in the Morphofunctional Assessment of Malnutrition in Patients Undergoing Incremental Versus Conventional Hemodialysis: A Comparative Study

**DOI:** 10.3390/medicina61091633

**Published:** 2025-09-09

**Authors:** Elena Jiménez Mayor, José C. De La Flor, Avinash Chandu Nanwani, Celia Rodríguez Tudero, Estefanya García-Menéndez, Raul Elias, Hemily Chimoy, Marco Dominguez Davalos, Michael Cieza Terrones, Francisco Valga, Jesús Hernández Vaquero

**Affiliations:** 1 Department of Nephrology, Hospital San Pedro de Alcántara, 10001 Cáceres, Spain; elena.jimenezm@salud-juntaex.es; 2 Department of Nephrology, Hospital Central de la Defensa Gómez Ulla, 28047 Madrid, Spain; jherva5@mde.es; 3 Department of Medicine and Medical Specialties, Faculty of Medicine, Alcala University, 28805 Madrid, Spain; 4 Health Sciences Doctoral Program, Faculty of Medicine, Alcala University, 28805 Madrid, Spain; 5 Department of Nephrology, Hospital General de Fuerteventura, 35600 Fuerteventura, Spain; achanan@gobiernodecanarias.org; 6 Department of Nephrology, Hospital Universitario de Salamanca, 37007 Salamanca, Spain; crodrigueztudero@usal.es; 7 Surgery Doctoral Program, Faculty of Medicine, University of Salamanca, 37007 Salamanca, Spain; 8 Department of Nephrology, Hospital Universitario Puerta de Hierro Majadahonda, 28222 Madrid, Spain; estefanialisset.garcia@salud.madrid.org; 9 Department of Nephrology, Hospital Cayetano Heredia, Lima 15002, Peru; raul.elias.c@upch.pe (R.E.); marco.dominguez.d@upch.pe (M.D.D.); 10 Department of Nephrology, Hospital Guillermo Almenara, Lima 15001, Peru; 2021031803@unfv.edu.pe; 11 Department of Engineering, Faculty of Science and Engineering, Peruana Cayetano Heredia University, Lima 15012, Peru; michael.cieza@upch.pe; 12 Department of Nephrology, Doctor Negrin University Hospital of Gran Canaria, 35010 Las Palmas de Gran Canaria, Spain; evalamae@gobiernodecanarias.org; 13 Department of Medicine, University Fernando Pessoa-Canarias, 35450 Santa Maria de Guia, Spain

**Keywords:** incremental hemodialysis, nutritional status, nutritional ultrasound, sarcopenia, bioelectrical impedance

## Abstract

*Background and Objectives*: Nutritional status is essential for outcomes in hemodialysis (HD) patients. Incremental HD (iHD) may help preserve residual renal function, but its effect on nutrition and body composition is unclear. Nutritional ultrasound (NUS) offers a non-invasive way to assess muscle and fat, complementing methods like BIA. This study compared nutritional status using morphofunctional assessment in patients on iHD versus conventional HD (cHD). *Material and Methods*: This single-center observational cross-sectional study included 74 stable adult HD patients (>3 months). Patients were stratified into iHD (n = 13; 1–2 sessions/week) and cHD (n = 61; 3 sessions/week). Evaluations included clinical and biochemical parameters, BIA, handgrip strength, nutritional scores and NUS assessed mass muscle of anterior quadriceps rectus femoris (QRF), supramuscular fat (SMF), subcutaneous adipose tissue (SAT), and preperitoneal visceral fat (PPVF). *Results*: Patients on iHD exhibited a more favorable nutritional and inflammatory profile, with a lower risk of malnutrition and a reduced prevalence of protein-energy wasting (PEW) syndrome. Although BIA failed to clearly differentiate between groups, NUS identified better preservation of SMF in iHD patients (8.3 ± 2.5 vs. 6.6 ± 2 mm; *p* = 0.009), as well as higher preperitoneal visceral fat thickness (1.9 ± 4.9 vs. 0.6 ± 0.3 cm; *p* = 0.04). There was also a trend toward greater muscle thickness in the iHD group, such as the Y-axis (9.5 ± 2 vs. 8.5 ± 2.3 mm; *p* = 0.17) and cross-sectional area muscle of rectus femoris (CS-MARF in cm^2^) (2.9 ± 0.6 vs. 2.6 ± 0.8 mm; *p* = 0.1) of anterior QRF, although without reaching statistical significance. *Conclusions*: These results highlight the value of NUS as a sensitive method for assessing nutritional status in HD patients, particularly within individualized strategies such as iHD, where it may provide key complementary information not captured by conventional methods.

## 1. Introduction

Chronic kidney disease (CKD) represents a growing public health concern, associated with a high rate of morbidity and mortality. As it progresses, many patients require renal replacement therapies such as hemodialysis (HD) [1]. The majority of patients with advanced CKD (ACKD) undergo a conventional thrice-weekly dialysis regimen to reach a minimum single-pool Kt/V greater than 1.2, independent of residual renal function (RRF) or individual preference [2].

Incremental hemodialysis (iHD) is a validated strategy for patients with preserved RRF, allowing initiation with one or two sessions per week [3]. Evidence suggests that over half of new patients with end-stage kidney disease (ESKD) have sufficient RRF to begin with iHD. The best outcomes with iHD therapy have been observed when 24 h urine urea clearance (KrU) exceeds 3 mL/min and 24 h urine output is >500 mL, although these thresholds are considered conservative [4].

IHD may potentially preserve RRF, which is associated with the clearance of middle and large uremic toxins, including those bound to plasma proteins, such as p-cresol. This contributes to better anemia control, reduced inflammation, improved blood pressure management, enhanced quality of life, and improved nutritional status [5]. Among these, nutritional improvement is particularly relevant, as malnutrition is relatively common in HD patients (prevalence ranging from 20% to 70%, depending on diagnostic criteria) and is linked to a higher risk of hospitalization, infections, refractory anemia, frailty, sarcopenia, and cardiovascular mortality [6]. In this context, morphofunctional assessment plays a central role in the comprehensive management of malnutrition in HD patients.

IHD, particularly in its once-weekly form, may be indicated in selected patients as part of a conservative strategy that includes structured nutritional management. This may involve an individualized low-protein diet (0.6 g/kg/day) combined with supplementation of essential amino acid ketoanalogues (0.1 g/kg/day), aiming to preserve RRF without compromising nutritional status. This approach is endorsed by the 2020 KDOQI guidelines [7], which advocate its use in patients with ACKD or those receiving renal replacement therapy (RRT), provided appropriate nutritional follow-up is ensured. Nonetheless, specific evidence regarding the nutritional impact of this modality remains limited, particularly when assessed through morphofunctional techniques. This is clinically relevant, as malnutrition and sarcopenia are common in this population and significantly affect clinical outcomes and quality of life [8].

Nutritional ultrasound (NUS) is an accessible, reproducible, and radiation-free technique for the direct assessment of muscle mass and preperitoneal visceral fat (PPVF). Although it remains operator-dependent and inherently subjective, when integrated with clinical, biochemical, and functional parameters, NUS provides valuable information that contributes to a comprehensive morphofunctional evaluation of nutritional status [9]. Anterior quadriceps rectus femoris (QRF) ultrasound assesses both the quantity and quality of muscle mass, while abdominal wall ultrasound primarily evaluates PPVF (cardiovascular risk), superficial subcutaneous fat (SSCF) tissue (energy reserve), and deep subcutaneous fat (DSCF) tissue (neuroendocrine regulation). This measurement provides insights into the patient’s metabolic and inflammatory environment, given the role of visceral fat in proinflammatory processes. Whereas subcutaneous adipose tissue (SAT), measured by the thickness of the anterior abdominal subcutaneous fat layer, is considered an indirect marker of the patient’s energy reserves [10]. Although its application in renal patients is expanding, specific literature on its use in iHD remains limited. The primary objective of this study is to compare nutritional status assessed by NUS of the anterior QRF mass muscle (anterior–posterior muscle thickness-Y-axis, and cross-sectional area of rectus femoris-CS-MARF) in patients undergoing iHD versus conventional hemodialysis (cHD). Secondary endpoints included the evaluation of fat tissue using NUS of supramuscular fat (SMF) and PPVF.

## 2. Materials and Methods

This was a cross-sectional observational study conducted in a cohort of 74 patients undergoing HD at a single center. Patients over 18 years of age, on HD for more than 3 months, clinically stable, and with signed informed consent were included. Exclusion criteria were active infections, liver disease, limb amputation, active malignancies, or inability to complete the morphofunctional malnutrition assessment.

This work represents a specific subanalysis of a previously published study by our group, focused on the morphofunctional nutritional assessment of HD patients [11]. For the present analysis, clinical, biochemical, anthropometric, bioelectrical impedance analysis (BIA), handgrip strength, functional test, nutritional scales and NUS variables were included, following the previously described protocol.

For this subanalysis, two groups were classified according to the dialysis regimen: cHD, with patients receiving three weekly sessions (n = 61), and iHD, with patients receiving one or two weekly sessions (n = 13). Assignment to iHD followed a clinical protocol based on RRF criteria, adapted of the Incremental Hemodialysis in Incident Patients (IHDIP) study by Deira et al. [12] and the study proposal by Kalantar-Zadeh et al. [13]. The criteria were as follows: for once-weekly sessions, KrU ≥ 4 mL/min/1.73 m^2^ and urine output ≥ 1000 mL/24 h were required. For twice-weekly sessions, KrU between 2 and 4 mL/min/1.73 m^2^ and urine output ≥ 500 mL/24 h were necessary, along with clinical stability and adequate volume control. In addition to these allocation criteria, baseline RRF assessment also included measurement of 24 h urine collection proteinuria, which was recorded in all iHD patients (n = 13) and in those cHD patients with preserved residual diuresis (n = 20), and incorporated into the comparative analysis.

Sociodemographic variables, comorbidities, dialysis vintage, vascular access type, RRF (KrU, 24 h urine output, and 24 h urine collection proteinuria) and dialysis adequacy parameters, systolic blood pressure (SBP), diastolic blood pressure (DBP), and interdialytic weight gain (IDWG) were collected.

### 2.1. Morphofunctional Assessment of Malnutrition Included

#### 2.1.1. Anthropometric Measurements

Weight in kilograms (kg), height in meters (m), and body mass index (BMI), calculated as weight/height × height (kg/m^2^). The waist circumference (WC) was measured at the midpoint between the last costal border and the iliac crest, at the navel level, without pressure, by applying the tape measure horizontally (cm). Triceps and suprailiac skinfold thicknesses (mm) were measured according to established recommendations by the International Society for the Advancement of Kinanthropometry (ISAK) protocol [14]. Anthropometric measurements were obtained by a single operator to avoid inter-observer variability.

#### 2.1.2. BIA

Measurement of appendicular skeletal muscle mass (Kg) (ASSM); appendicular skeletal muscle mass index (Kg/m^2^) (ASMMI); phase angle (°) (PA); Visceral fat area (cm^2^) (VFA); fat-free mass (kg) (FFM); lean body mass (Kg) (LBM); body fat mass (kg) (BFM); total body water (L) (TBW); intracellular water (L) (ICW); extracellular water (L) (ECW). Nevertheless, this methodology has certain limitations, such as the requirement for specific consumables and contraindications in patients with pacemakers. In addition, among individuals with ESRD undergoing RRT, hydration status may lead to an overestimation of ASMM, representing a major limitation in those with irregular fluid balance. To minimize this effect, BIA is performed at least 30 min after the completion of the HD session to allow sufficient redistribution of body fluids across compartments [7,15].

#### 2.1.3. Handgrip Strength (HGS)

Assessed using dynamometry (mean of three measurements with the dominant hand, excluding the fistula arm), with values adjusted for age and sex. The assessment was conducted with the patient in a seated position, keeping the dominant or non-fistula hand at a right angle to the body. Reduced handgrip strength was defined as <27 kg in men and <16 kg in women, in accordance with the 2019 European Working Group on Sarcopenia in Older people (EWGSOP2) consensus recommendation [16].

#### 2.1.4. Nutritional and Frailty Scales

Malnutrition Inflammation Score (MIS) [17], 7- points Subjective Global Assessment (7p-SGA) scale [18], Malnutrition Screening Tool (MST) [19], Protein-Energy Wasting (PEW) and confirmed sarcopenia in accordance with the 2019-EWGSOP2 [16]. Frailty was evaluated using the FRAIL scale [20].

#### 2.1.5. Functional Physical Performance Variable

The Short Physical Performance Battery (SPPB) was utilized to evaluate functional performance [21].

#### 2.1.6. Nutritional Ultrasound (NUS)

A structured protocol was applied for ultrasound-based body composition assessment across two compartments (muscle and adipose tissue). We followed the protocol published in a previous work of our group [11].

- Anterior QRF ultrasound: The variables measured to assess muscle mass were anterior–posterior muscle thickness (Y-axis in mm), adjusted for height (Y-axis/h in mm/m^2^) transversal muscle thickness (X-axis in mm), supramuscular fat (SMF in mm) and cross-sectional muscle area of rectus femoris (CS-MARF in cm^2^). The CS-MARF was standardized by height [MARF (cm^2^)/(height × height) (m^2^)], which was named the muscle area rectus femoris index (MARFI_h_ in cm^2^/m^2^).
- PPVF and abdominal subcutaneous fat: An imaginary line was drawn connecting the xiphoid process and the umbilicus. At the midpoint of this line, images of total transverse subcutaneous fat (SSCF in cm plus DSCF in cm) and PPVF were acquired, with the transducer positioned perpendicular to the longitudinal axis.

We followed the protocol published in a previous work of our group [11]. The anterior QRF NUS is accessible, highly reproducible, and sensitive to changes in skeletal muscle mass, allowing an indirect assessment of sarcopenia. NUS-derived cut-off values for sarcopenia were Y-axis ≤ 8 mm, Y-axis/height ≤ 2.9 mm/m^2^, CS-MARF ≤ 2.4 cm^2^, and MARFI_h_ ≤ 0.9 cm^2^/m^2^ [11]. To reduce inter- and intra-observer variability, all NUS measurements were conducted by the same trained operator, following a standardized protocol as described above, and always on the same day of the week (the middle day for conventional thrice-weekly HD and the last day of the week for iHD) to ensure consistent conditions. For each assessment, three measurements were taken, and the mean value was used for analysis to minimize random error and enhance reliability. Similarly to BIA, NUS measurements can be affected by tissue hydration, particularly in edematous patients, although this influence is less pronounced than with BIA. To account for this, all assessments were performed post-dialysis. Examinations were conducted with the patient in the supine position and under muscle relaxation.

#### 2.1.7. Analytic Variables

Laboratory data included blood count parameters, iron kinetics, electrolytes, bone mineral metabolism, lipid profile laboratory data included hemoglobin, lymphocytes, ferritin, transferrin saturation index (TSI), total proteins, albumin, pre-albumin, C-reactive protein (CRP), serum sodium (sNa+), serum chloride (sCl−), serum potassium (sK+), bicarbonate, total serum calcium (sCa++), serum phosphorus (sP), serum intact parathyroid hormone (iPTH), 25OH-Vitamin D, total cholesterol, high-density lipoprotein (HDL), low-density lipoprotein (LDL), non-HDL, triglycerides, serum urea (sU) and serum creatinine (sCr).

### 2.2. Statistical Analysis

Statistical analyses were performed using Stata 16. Quantitative variables were expressed as mean (standard deviation, SD) or median (interquartile range, IQR), depending on distribution normality, and compared between groups using Student’s *t*-test or Mann–Whitney U test as appropriate. Categorical variables are presented as frequencies or percentages and compared using chi-square or Fisher’s exact tests. A *p*-value < 0.05 was considered statistically significant.

Between-group differences were further quantified using effect sizes: Hedges’ g for continuous variables (given the small sample size). Baseline balance was assessed using both conventional hypothesis testing (*p*-values). For secondary analyses, multiple comparisons were controlled using the Bonferroni correction to adjust for the risk of type I error. Correlation analyses were performed using Pearson’s r or Spearman’s rho as appropriate. Partial correlations adjusted for age and body mass index (BMI) were reported where relevant. Multivariable linear regression was used to assess the association between dialysis modality (iHD vs. cHD) and Y-axis (mm) or CS-MARF (cm^2^), adjusting for age, sex, BMI, and diabetes mellitus. Robust standard errors were applied, and regression coefficients (β), 95% confidence intervals, and *p*-values were reported. Given the limited sample size, the number of covariates included in multivariable models was restricted to avoid overfitting. All tests were two-sided with α = 0.05.

## 3. Results

74 patients were enrolled, with 61 in the cHD group and 13 in the iHD group. Regarding baseline clinical and laboratory characteristics, patients in the iHD group had a shorter dialysis vintage (21.1 vs. 42.5 months; *p* = 0.004) and higher serum magnesium levels (*p* = 0.02), as well as significantly lower concentrations of blood urea nitrogen (*p* = 0.004), serum urea (*p* = 0.004), serum chloride (*p* = 0.005), and normalized protein catabolic rate (nPCR) (*p* = 0.0004), compared to those in the cHD group. No significant differences were observed in serum prealbumin (26.39 ± 6.24 vs. 26.86 ± 8.89 mg/dL, *p* = 0.86) or albumin (3.20 ± 0.51 vs. 3.38 ± 0.47 g/dL, *p* = 0.24) levels between groups (Table 1). A comprehensive analysis of baseline clinical and laboratory variables is presented in Table 1 and Table 2. As expected, patients in the iHD group had significantly higher KrU values, 24 h urine output, and 24 h urine collection proteinuria compared to the cHD group, consistent with their better preserved RRF. It should be noted that RRF parameters were assessed in all iHD patients and in the 20 cHD patients who maintained RRF (Table 1).

**Figure 1:**
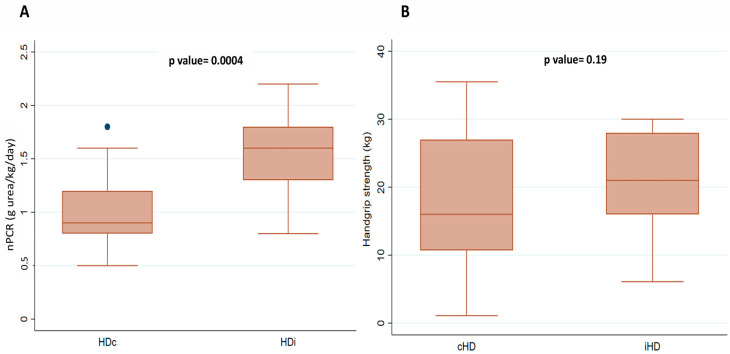
Comparison of nPCR (**A**) and handgrip strength (**B**) between patients on cHD and iHD. Abbreviations: iHD, incremental hemodialysis modality; cHD, Conventional hemodialysis; nPCR, normalized protein catabolic rate.

Regarding clinical and functional nutritional indicators, nPCR a reference marker of protein metabolism and a key nutritional indicator in HD patients—was significantly higher in the iHD group (1.55 ± 0.41 g/kg/day) compared to the cHD group (1.0 ± 0.31; *p* = 0.0004). HGS was slightly higher in the iHD group, but the difference was not statistically significant (*p* = 0.19). The proportion of patients with reduced HGS did not differ significantly between groups, see Figure 1 and Table 3.

Regarding malnutrition and inflammation scales, the MIS was significantly lower in the iHD group (5.6 ± 4.9 vs. 8.1 ± 3.5; *p* = 0.04), and the percentage of patients with MIS > 8 was markedly lower (7.7% vs. 47.5%; *p* = 0.008). Additionally, the MST was lower in the iHD group (7.7% vs. 24.6%; *p* = 0.18), as was the prevalence of malnutrition according to the 7p-SGA (30.8% vs. 54.1%; *p* = 0.13). Based on the PEW score, iHD patients also showed a lower prevalence (23.1% vs. 49.2%; *p* = 0.09), further supporting this favorable trend. The prevalence of sarcopenia according to the 2019 EWGSOP2 consensus recommendation [11], was lower in the iHD vs. cHD group (15.4% vs. 37.3%, *p* = 0.12) without reaching significance. Frailty status was also assessed using the FRAIL scale, revealing a significantly lower prevalence of frailty in the iHD group (7.7%) compared to the cHD group (45.9%) (*p* < 0.04). A complete summary of results is presented in Table 3.

Regarding body composition assessed by BIA, no significant differences were found between groups in terms of hydration and body composition parameters, as summarized in Table 4.

**Figure 2:**
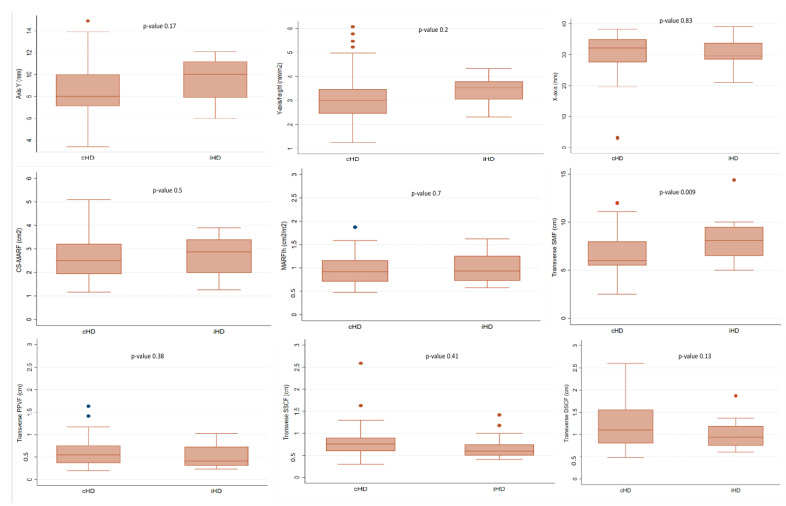
Box plots of comparison of nutritional ultrasound parameters between conventional and incremental hemodialysis patients. Abbreviations: iHD, incremental hemodialysis modality; cHD, Conventional hemodialysis; SMF, Supramuscular fat; PPVF, preperitoneal visceral fat; DSCF, deep subcutaneous fat; SSCF, superficial subcutaneous fat; CS-MARF, cross-section muscle area of the rectus femoris; MARFI^h^, muscle area rectus femoris index ajusted height^2^.

Regarding NUS, no significant differences were observed in anterior QRF parameters, including the Y-axis (9.5 vs. 8.5 mm; *p* = 0.17), or CS-MARF (2.9 vs. 2.6 cm^2^, *p* = 0.1) as primary endpoints. For secondary endpoints, transverse SMF was higher in iHD patients, (8.3 vs. 6.6 cm; *p* = 0.009) while no significant differences were observed in PPVF. After Bonferroni correction for two secondary tests, SMF remained statistically significant (Table 5 and Figure 2).

**Figure 3:**
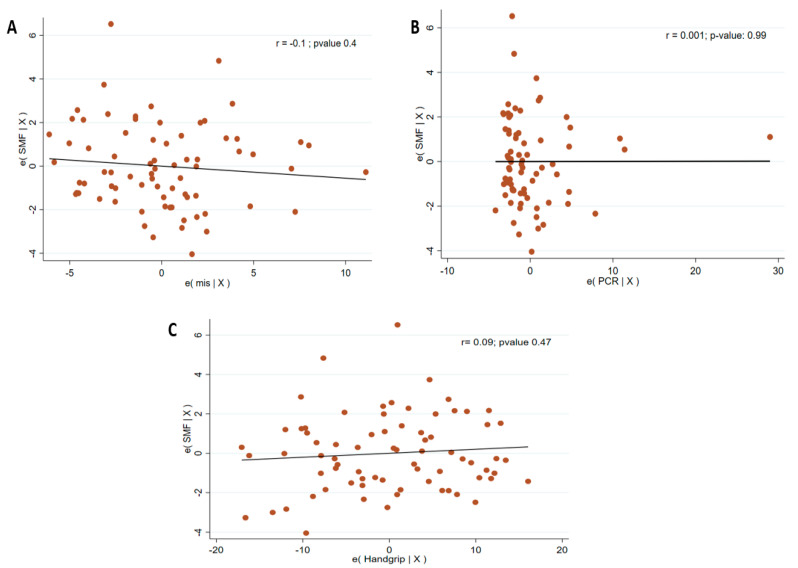
Partial correlation between SMF and MIS (**A**)/CRP (**B**)/Handgrip (**C**), adjusted for age and BMI. Abbreviations: SMF, sumpramuscular fat; RCP, CRP, C-reactive protein; BMI, body mass index.

Effect size for Y-axis and CS-MARF between iHD and cHD groups was small (Hedges’ g = 0.17, 95% CI −0.42 to 0.76; Hedges’ g = 0.20, 95% CI −0.39 to 0.80), consistent with the non-significant difference observed in the unadjusted comparison (*p* = 0.17). Among the secondary endpoints, SMF was significantly higher in the iHD group, with a large effect size (Hedges’ g = 0.82, 95% CI 0.21 to 1.42). No significant association was found between SMF and MIS, CRP, or HGS after adjusting for age/BMI (Figure 3). PPVF did not differ significantly between groups and the effect size was negligible (Hedges’ g close to 0). In multivariable linear regression adjusted for age, sex, BMI, and diabetes mellitus, iHD was not significantly associated with Y-axis (β = 0.82; 95% CI −0.31 to 1.95; *p* = 0.151). Older age (β = −0.031; 95% CI −0.060 to −0.001; *p* = 0.040) and higher BMI was associated with Y-axis (β = 0.202; 95% CI 0.098–0.306; *p* < 0.001); sex and diabetes status were not significantly associated.

## 4. Discussion

To the best of our knowledge, this is the first real-world study to assess nutritional status through morphofunctional evaluation using nutritional ultrasound (NUS) in HD patients, specifically comparing the iHD and cHD modalities. Our findings suggest a trend toward better preservation of muscle mass in the iHD group, as assessed by NUS, with a non-significant difference in Y-axis and CS-MARF of the anterior QRF between groups. No significant difference in PPVF was observed. However, the iHD group presented a higher SMF, a difference that was statistically significant and accompanied by a large effect size, which may be consistent with a less catabolic metabolic environment in these patients.

The growing number of patients with CKD on HD [2] necessitates consideration of additional factors such as sarcopenia, malnutrition, and frailty—highly prevalent comorbidities in patients undergoing RRT as HD. These conditions are driven by factors such as uremic toxin accumulation, metabolic acidosis, vitamin D deficiency, insulin resistance, hyperparathyroidism, proteinuria, amino acid losses during dialysis, and chronic inflammation, both of which have been shown in several meta-analyses to be important predictors of cardiovascular events [16,22]. Duarte et al. estimated a global prevalence of sarcopenia of 24.5%, which increases to 26.2% in dialysis patients [23]. Mariño et al. reported a coexistence of sarcopenia and frailty in 17.1% of HD patients in Peru [24]. In the overall population of the original study conducted by our group, we found a sarcopenia prevalence of 28.4%, frailty in 34.5%, and both conditions in 16.5% of patients. Initially, no stratified analysis was performed based on the dialysis modality, so differences between cHD and iHD had not been previously described. In the present subanalysis, when stratifying the results according to treatment modality, a prevalence of sarcopenia of 15.4% and frailty of 7.7% was observed in patients undergoing iHD, compared to 37.7% and 45.9%, respectively, in those on cHD. Despite a higher mean age in the iHD group (75.5 vs. 72.6 years), functional, nutritional, and inflammatory indicators were more favorable in this group. This finding acquires clinical relevance, as it suggests that the incremental regimen could attenuate age-associated deterioration, possibly due to a lower dialysis burden and better preservation of RRF [2,4,13,25]. No previous studies have been found that specifically analyze these aspects in patients on iHD, reinforcing the pioneering nature of this study.

Comparison between BIA and NUS measurements revealed comparable estimates of muscle mass, although the degree of agreement varied by parameter. For instance, ASMM values derived from BIA were in line with muscle mass estimations obtained from anterior QRF NUS measurements, SAT assessments showed less concordance between the two modalities. These findings suggest that BIA and NUS may offer complementary perspectives whereas in evaluating body composition, with NUS providing anatomical detail and BIA offering an integrated analysis of fluid and tissue compartments. This aligns with previous reports highlighting the potential for combining these methods to enhance nutritional and functional assessment in patients undergoing hemodialysis.

A key principle underlying iHD is the preservation of RRF, which is linked to reduced inflammation, improved metabolic stability, and better nutritional status [2,15]. In this context, the IHDIP clinical trial, registered at ClinicalTrials.gov (NCT03239808), is currently evaluating the structured implementation of iHD in routine clinical practice. This protocol is based on the findings of the IHDIP study [12].

In our cohort, patients undergoing iHD presented a significantly lower MIS and a reduced proportion of patients with MIS ≥ 8 points (7.7% vs. 47.5%; *p* = 0.008), consistent with a more favorable nutritional and inflammatory profile, although causality cannot be inferred from these cross-sectional data. Additionally, the prevalence of PEW > 2 points was lower in the iHD group (23.1% vs. 49.2%), although it did not reach statistical significance. These findings are consistent with those reported by Caria et al. [25], who demonstrated that an incremental dialysis regimen, combined with individualized dietary counseling, improved nutritional parameters such as serum albumin and transferrin. This evidence is further supported by the study of Locatelli et al. [26], who highlighted the benefits of low-protein diets not only in preserving nutritional status but also in maintaining RRF in patients on iHD. These prior findings align with the associations observed in our study.

Regarding the assessment of muscle strength, HGS was higher in the iHD group, without reaching statistical differences. The BIA parameters studied showed no relevant differences between groups in estimated muscle mass or phase angle, underscoring its limitations in assessing LBM in HD patients and supporting the potential role of complementary methods such as NUS [6]. In contrast, the most relevant findings were obtained by NUS: the transverse SMF (mm) was significantly greater in the iHD group. Although no statistically significant differences were found in the anterior QRF thickness along the Y-axis or in its CS-MARF, a favorable trend was observed in the iHD group. In multivariate analysis, the initial HD technique was not significantly associated with the Y-axis of anterior QRF-NUS after adjusting for BMI and age, suggesting that demographic and anthropometric characteristics have a greater impact on muscle morphology than the type of HD. Despite a finding, given the cross-sectional design of the study and the limited sample size, it is not possible to establish a causal relationship or determine its long-term clinical relevance. These findings underscore the utility of NUS in detecting morphostructural alterations not evident through BIA or indirect functional methods such as HGS. In this regard, previous studies, such as that by Bahşi et al., have demonstrated that measurements like visceral fat thickness can correlate with muscle status and predict sarcopenia [27].

Although serum albumin and prealbumin are commonly used as nutritional biomarkers, their levels in our cohort did not differ significantly between groups. Albumin is a well-established marker that reflects both nutritional status and chronic inflammation and is closely associated with outcomes in hemodialysis patients. Previous studies have shown that hypoalbuminemia is linked to increased morbidity and mortality in this population [28]. This finding is in line with previous evidence suggesting that these parameters may be influenced by inflammation, hydration status, and comorbidities, limiting their discriminatory power in CKD populations. Similarly, a meta-analysis comparing incremental versus conventional HD reported no significant differences in serum albumin between both modalities [29]. These findings align with our own observations, where albumin values did not differ significantly between groups for albumin. However, the Chinese Clinical Practice Guidelines for Nutritional Assessment and Monitoring of Adult ICU Patients, emphasize that serum albumin, although influenced by inflammation and fluid status, remains a useful parameter for longitudinal monitoring when interpreted alongside comprehensive nutritional assessment tools [7].

In nephrology practice, nutritional assessment is often based on indirect tools such as subjective scales (SGA, MIS), PEW criteria, or strength tests like dynamometry (HGS). Although BIA is widely used due to its low cost, it can be influenced by hydration status. In contrast, NUS provides a direct anatomical evaluation of muscle and adipose tissue without interference from fluid status and has demonstrated good correlation with computed tomography or dual-energy X-ray absorptiometry (DEXA), considered the gold standard [9,16].

One of the primary limitations of our study are its single-center cross-sectional design, which limits the generalizability of the findings and precludes establishing causal relationships, the unbalanced group size, which reduces the statistical power to detect subtle differences and may have influenced the robustness of between-group comparisons, and the fact that patients in the iHD group had a significantly shorter dialysis vintage, which could reflect a better preserved RRF and nutritional status at baseline, potentially confounding the observed differences. Additionally, no matching was performed between groups, so the iHD and cHD cohorts are not fully comparable in potentially influential variables such as age, comorbidities, or dialysis vintage. Although consistent clinical criteria were applied for assigning patients to iHD, the influence of unmeasured confounding factors cannot be ruled out, as this modality is usually selected for patients with better functional status and a more favorable metabolic profile. Nevertheless, the concordance of our results with earlier studies, the use of innovative morphofunctional methodology, and the direct comparison between dialysis modalities in a real-world clinical setting strengthen the relevance of the results obtained. Accordingly, our results should be interpreted as hypothesis-generating rather than definitive.

Finally, we consider that NUS should be systematically integrated into the nutritional assessment of hemodialysis patients, particularly in personalized strategies such as iHD, where its sensitivity to detect early structural alterations may have a significant clinical impact. As a practical recommendation, NUS could be implemented on a monthly or bimonthly basis within a simple standard operating procedure (SOP). Tracking trends in SMF and PPVF, in conjunction with MIS and HGS, may allow early identification of nutritional risk, particularly in patients where BIA is sensitive to changes in fluid status, such as those with significant interdialytic weight gain or edema. This work could lay the groundwork for future prospective trials evaluating the utility of NUS as a sensitive and effective marker in the monitoring of individualized hemodialysis programs.

## 5. Conclusions

Patients undergoing iHD presented a nutritional profile that was more favorable than that of patients on cHD, particularly in NUS-derived parameters. In this observational cross-sectional study, NUS proved to be a sensitive and feasible technique to detect morphofunctional alterations that may go unnoticed with traditional tools such as BIA or functional and strength tests. Considering its accessibility, cost-effectiveness, and reproducibility, the systematic integration of NUS into the monitoring of hemodialysis patients, both cHD and iHD, appears justified and could enhance the early detection of nutritional risk. These findings also support the development of future longitudinal studies to evaluate whether the potential benefits observed with iHD translate into improved clinical and functional outcomes in the medium and long term. While our results should be interpreted with the inherent caution of an observational study, the incorporation of techniques such as NUS may represent a significant step forward in optimizing nutritional status in dialysis patients and promoting more personalized, effective care focused on preserving functionality.

## Figures and Tables

**Table 1:** Sociodemographic, Anthropometrical, causes of CKD and Hemodialysis parameters.

Variable	cHD	iHD	*p* Value
	Demographic and body measurement parameters		
Age (years), mean (SD)	72.6 (15.8)	75.5 (14.5)	0.526
Sex (Men), n (%)	43 (70.5%)	9 (69.2%)	1.000
Weight (kg)	67.63 (12.71)	70.9 (10.45)	0.34
Height (m)	1.66 (0.1)	1.66 (0.1)	0.99
BMI (kg/m^2^), mean (SD)	24.78 (4.4)	25.75 (3.27)	0.37
Body surface area (m^2^), mean (SD)	1.73 (0.19)	1.77 (0.16)	0.50
Waist circumference (cm), mean (SD)	78.6 (12.9)	73.9 (21.7)	0.463
Triceps skinfold (mm), mean (SD)	12.3 (6.4)	12.5 (4.9)	0.934
Suprailiac skinfold (mm), mean (SD)	16.5 (7.4)	18.0 (10.6)	0.628
COPD, n (%)	17 (27.9%)	6 (46.2%)	0.206
Ischemic heart disease, n (%)	31 (50.8%)	6 (46.2%)	1.000
Secondary hyperparathyroidism, n (%)	60 (98.4%)	13 (100.0%)	1.000
Oncologic disease, n (%)	17 (27.9%)	2 (15.4%)	0.494
Depression, n (%)	6 (9.8%)	0 (0.0%)	0.583
Gastrointestinal disorders, n (%)	16 (26.2%)	1 (7.7%)	0.275
	Causes of CKD		
Diabetic kidney disease, n (%)	22 (36.1%)	4 (30.8%)	0.200
ADPKD, n (%)	4 (6.6%)	0 (0%)	0.547
Glomerular disease, n (%)	6 (15.4%)	2 (15.4%)	1.000
	Hemodialysis parameters		
HD vintage (months), mean (SD)	42.5 (34.0)	21.1 (19.5)	0.004
Dry weight (kg), mean (SD)	67.6 (12.7)	70.9 (10.5)	0.327
IDWG (kg), mean (SD)	2.18 (0.7)	1.70 (0.6)	0.026
Kt/V urea, mean (SD)	1.6 (0.2)	1.7 (0.3)	0.160
KT (L), mean (SD)	51.4 (6.4)	54.9 (5.5)	0.060
nPCR (g urea/kg/day), mean (SD)	1.0 (0.3)	1.55 (0.4)	0.0004
QB (mL/min), mean (SD)	337.2 (22.3)	325.4 (13.5)	0.07
APF (mL/min)	177.1 (34.0)	152.8 (40.8)	0.063
VPF (mL/min)	163.8 (21.2)	155.3 (28.1)	0.316
SBP (mmHg)	126.7 (25.8)	142.2 (16.5)	0.011
DBP (mmHg)	83.6 (59.3)	68.9 (12.4)	0.322
	Renal Residual Function		
KrU (mL/min/1.73 m^2^), mean (SD)	1.2 (0.8)	4.1 (1.1)	<0.001
24 h urine output (mL)	420 (280)	1150 (420)	<0.001
Proteinuria (mg), mean (SD)	105 (80)	815 (260)	<0.001
	Vascular access type		
Arteriovenous fistula (%)	37.7%	62.3%	1.000
Tunneled catheter (%)	62%	38%	1.000

Abbreviations: SD, standard desviation; %, percent; Kg, kilograms, cHD, conventional hemodialysis; iHD, incremental hemodialysis; BMI, body mass index; COPD, chronic obstructive pulmonary disease; CKD, chronic kidney disease; ADPKD, autosomal dominant polycystic kidney disease; HD vintage, time on hemodialysis; IDWG, interdialytic weight gain; Kt/V, dialysis adequacy index based on urea clearance; KT, total urea clearance; nPCR, normalized protein catabolic rate; QB, blood flow rate; APF, arteriovenous pump flow; VPF, venous pump flow; SBP, systolic blood pressure; DBP, diastolic blood pressure; KrU, urea clearance.

**Table 2:** Baseline clinical and laboratory characteristics of patients on conventional and incremental hemodialysis.

	cHD	iHD	*p* Value
Patients (N)	61	13	
Hemoglobin (g/L), mean (SD)	11.27 (1.67)	10.98 (1.1)	0.44
Fe (mg/dL), mean (SD)	72.34 (34.06)	58.08 (27.35)	0.12
TSI (%), mean (SD)	33.46 (5.5)	28.31 (12.61)	0.21
Transferrin (mg/dL), mean (SD)	171.36 (30.7)	160.08 (20.12)	0.11
Ferritin (ng/mL), mean (SD)	741.9 (560.6)	937.69 (357.53)	0.12
Calcium (mg/dL), mean (SD)	8.84 (0.69)	8.92 (0.74)	0.71
Phosphorus (mg/dL), mean (SD)	4.26 (1.59)	4.42 (1.15)	0.67
25OHD (ng/mL), mean (SD)	26.46 (14.55)	20.88 (9.33)	0.09
PTH (pg/mL), mean (SD)	254.08 (206.39)	275.87 (220.2)	0.75
Magnesium (mg/dL), mean (SD)	2.14 (0.29)	2.35 (0.27)	0.02
Total Cholesterol (mg/dL), mean (SD)	130.92 (32.3)	125.0 (29.61)	0.53
HDL (mg/dL), mean (SD)	49.11 (19.17)	44.62 (11.13)	0.26
LDL (mg/dL), mean (SD)	62.08 (25.68)	59.85 (23.4)	0.76
non-HDL (mg/dL), mean (SD)	81.8 (28.37)	80.38 (23.89)	0.85
Triglycerides (mg/dL), mean (SD)	122.44 (71.23)	124.69 (55.18)	0.90
C-reactive protein (mg/L), mean (SD)	2.37 (4.92)	1.75 (4.04)	0.63
Lymphocytes (10^3^/μL), mean (SD)	1.09 (0.47)	1.22 (0.61)	0.49
Sodium (mmol/L), mean (SD)	137.93 (3.36)	139.23 (3.37)	0.22
Potassium (mmol/L), mean (SD)	4.58 (0.74)	4.39 (1.01)	0.53
Chloride (mmol/L), mean (SD)	101.1 (3.54)	104.38 (3.38)	0.005
Bicarbonate (mEq/L), mean (SD)	23.31 (2.31)	23.05 (2.96)	0.76
Urea (mmol/mL), mean (SD)	108.49 (43.81)	169.38 (62.41)	0.004
BUN (mmol/mL), mean (SD)	50.63 (20.45)	79.05 (29.12)	0.004
Creatinine (g/dL), mean (SD)	6.01 (2.02)	6.2 (2.97)	0.82
Total proteins (g/dL), mean (SD)	6.38 (0.62)	6.59 (0.51)	0.21
Prealbumin (mg/dL), mean (SD)	26.39 (6.24)	26.86 (8.89)	0.86
Albumin (g/dL), mean (SD)	3.2 (0.51)	3.38 (0.47)	0.24
Cr/BSA (mg/dL/m^2^)	3.49 (1.24)	3.53 (1.68)	0.93

Abbreviations: SD, Standard deviation; cHD; Conventional hemodialysis; iHD, Incremental hemodialysis; Fe, Iron; TSI, Transferrin Saturation Index; 25OHD, Vitamin D; PTH, Parathyroid hormone; HDL, High-density lipoprotein; LDL, Low-density lipoprotein; non-HDL, Non-high-density lipoprotein cholesterol; BUN, Blood urea nitrogen; Cr/BSA, Creatinine/body surface area ratio.

**Table 3:** Muscle strength, functional performance and nutritional screening scores.

	cHD	iHD	*p* Value
**Patients (N)**	61	13	
**Muscle strength**			
Handgrip strength (HGS) (kg), mean (SD)	17.6 (8.8)	21.1 (7.7)	0.19
Reduced HGS, n (%)	41 (67.2)	7 (53.9)	0.4
**Functional performance**			
SPPB (points)	8.6 (2.1)	8.9 (2.2)	0.2
Low performance (SPPB ≤ 8), n (%)	18 (29.5)	3 (23.07)	0.42
**Scales**			
MIS (points)	8.1 (3.5)	5.6 (4.9)	0.04
MIS > 8 points, n (%)	47.5	7.7	0.008
MST > 2 points, n (%)	24.6	7.7	0.18
7–points SGA scale (Mild–moderate–severely malnourished), n (%)	54.1	30.08	0.13
PEW > 2 points, n (%)	49.2	23.1	0.09
FRAIL scale, (score ≥ 3 points), n (%)	28 (45.9)	1 (7.7)	0.04
SARCOPENIA (2019-EWGSOP2), n (%)	23 (37.7)	2 (15.4)	0.12

Abbreviations: iHD, incremental hemodialysis modality; cHD, Conventional hemodialysis; SD, Standard deviation; PEW, Protein-Energy Wasting, Defined as ≥2 criteria present; MIS, Malnutrition Inflammation Score; MST, Malnutrition Screening Tool; SGA, Subjective Global Assessment; FRAIL, fatigue, resistance, ambulation, illnesses, and loss.; Reduced HGS, according to the 2019 EWGSOP2 consensus recommendation.

**Table 4:** Hydration and body composition parameters by bioimpedance analysis (BIA).

	cHD	iHD	*p* Value
Patients (N)	61	13	
ASMM (kg), mean (SD)	18.1 (4.5)	18.2 (4.3)	0.95
ASMMI (kg/m^2^), mean (SD)	6.5 (1.2)	6.6 (1.2)	0.88
PA (°), mean (SD)	4.8 (1.5)	4.8 (1.1)	0.92
VFA (cm^2^), mean (SD)	92.6 (59.9)	93.3 (36.9)	0.97
FFM (kg), mean (SD)	47.3 (11.6)	49.3 (9.4)	0.57
LBM (kg), mean (SD)	44.8 (10.5)	44.3 (10.8)	0.88
BFM (kg), mean (SD)	20.6 (10.1)	21.6 (7.3)	0.73
TBW (L), mean (SD)	35.2 (8.1)	34.8 (7.9)	0.86
ICW (L), mean (SD)	22.2 (8.9)	21.9 (3.9)	0.9
ECW (L), mean (SD)	14.1 (3.9)	14.4 (3.3)	0.76

Abbreviations: iHD, incremental hemodialysis modality; cHD, Conventional hemodialysis; BIA, bioimpedance analysis; ASMM, appendicular skeletal muscle mass; ASMMI, appendicular skeletal muscle mass index; PA, phase angle; Visceral fat area, VFA; FFM, fat-free mass; LBM, lean body mass; BFM, body fat mass; TBW: total body water; ICW: intracellular water; ECW: extracellular water.

**Table 5:** Comparison of nutritional ultrasound parameters between conventional and incremental hemodialysis patients.

	cHD	iHD	*p* Value
N	61	13	
Y-axis (mm), mean (SD)	8.5 (2.3)	9.5 (2)	0.17
X-axis (mm), mean (SD)	30.4 (6.8)	30.9 (5.3)	0.83
Y-axis/height (mm/m^2^), mean (SD)	3.1 (1.0)	3.4 (0.6)	0.2
Y-axis/BSA (mm/m^2^), mean (SD)	2.9 (0.9)	3.0 (0.6)	0.5
CS-MARF (cm^2^), mean (SD)	2.6 (0.8)	2.9 (0.6)	0.1
MARFI_h_ (cm^2^/m^2^), mean (SD)	0.96 (0.32)	1.0 (0.31)	0.66
Transverse SMF (cm) mean (SD)	6.6 (2)	8.3 (2.5)	0.009
Transverse PPVF (cm), mean (SD)	0.6 (0.3)	0.5 (0.3)	0.38
Transverse DSCF (cm) mean (SD)	1.2 (0.5)	1 (0.4)	0.13
Transverse SSCF (cm), mean (SD)	0.8 (0.4)	0.7 (0.3)	0.41

Abbreviations: SD, standard desviation; iHD, incremental hemodialysis modality; cHD, Conventional hemodialysis; SMF, Supramuscular fat; PPVF, preperitoneal visceral fat; DSCF, deep subcutaneous fat; SSCF, superficial subcutaneous fat; Y-axis, X-axis, Y-axis height, Y-axis/BSA, ultrasound-derived muscle cross-sectional diameters adjusted for BSA (body surface area) (mm/m^2^); CS-MARF, cross-section muscle area of the rectus femoris; MARFIh, muscle area of the rectus femoris index adjusted to height.

## Data Availability

This study is a subanalysis of a previously published dataset generated by our research group. For the present work, data were reanalyzed to address the specific objectives of this study. Owing to patient privacy and ethical considerations, the dataset is not publicly available; however, it can be requested from the corresponding author (J.C.D.L.F., jflomer@mde.es) upon reasonable request and with permission from the Ethics Committee. All figures and tables are original creations by the authors.
